# Trends in mortality with chronic obstructive pulmonary disease as the underlying cause and chronic kidney disease as a contributing cause among US adults, 1999–2020: a CDC WONDER analysis

**DOI:** 10.1186/s12890-026-04499-2

**Published:** 2026-07-13

**Authors:** Chengying Zhu, Guoxin Zhang

**Affiliations:** 1 Department of Respiratory Medicine, Nanjing Lishui People’s Hospital (Zhongda Hospital Lishui Branch), Southeast University, No.86,Chongwen Road,Lishui District, Nanjing, Jiangsu 211200 China; 2 Department of Geriatrics, Nanjing Qixia District Hospital, 28 Yaojia Road, Nanjing, Jiangsu 210046 China

**Keywords:** Chronic obstructive pulmonary disease, CDC WONDER database, Chronic kidney disease, Mortality trends, Health disparities

## Abstract

**Background:**

Chronic obstructive pulmonary disease (COPD) and chronic kidney disease (CKD) frequently coexist, yet mortality trends in patients with both conditions remain poorly characterized.

**Methods:**

Using the CDC WONDER Multiple Cause of Death database, we analyzed death certificates from 1999 to 2020 for adults aged ≥ 25 years with COPD (ICD-10: J40-J44) as the underlying cause and CKD (N18) as a contributing cause. Age-adjusted mortality rates (AAMRs) per 100,000 population were calculated using 2000 US standard population. Trends were assessed via Joinpoint regression, with stratifications by sex, race/ethnicity, age, urbanization, census regions, and state.

**Results:**

Deaths increased from 1,405 in 1999 to 5,277 in 2020. The AAMR increased from 0.79 in 1999 to 1.96 in 2020. The overall average annual percent change (AAPC) was + 4.71% (95% CI: 3.06–6.38). Mortality rates were higher in males (AAMR: 2.41) than females (AAMR: 1.65), though females had faster increases (AAPC + 5.86% vs. + 3.42%). The 85 + age group had the highest rates (27.98 per 100,000 population) and fastest growth (APC + 6.43%). non-Hispanic (NH) White individuals exhibited the steepest increase (AAPC + 5.25%), while NH Black individuals had no significant trend (AAPC − 0.22%). Nonmetropolitan areas had higher AAMRs (2.68) compared to metropolitan areas (1.82). Regionally, the Midwest recorded the highest AAPC (+ 5.32%) and 2020 AAMR (2.42). State-level AAPCs ranged from 1.5% to 7.0%.

**Conclusion:**

Rising COPD-related mortality among adults with CKD highlights a need for integrated pulmonary-renal care, targeted interventions for high-risk populations, and policies addressing rural healthcare access and environmental risk factors. These estimates reflect deaths certified with COPD as the underlying cause and CKD as a contributing cause, and therefore do not represent mortality among all individuals with coexisting COPD and CKD.

**Supplementary Information:**

The online version contains supplementary material available at https://doi.org/10.1186/s12890-026-04499-2.

## Introduction

Chronic obstructive pulmonary disease (COPD) affects over 15 million US adults and ranks as the fourth leading cause of death nationally [1, 2], with mortality rates continuing to rise despite declining smoking prevalence [3]. Chronic kidney disease (CKD) affects approximately 37 million Americans, with prevalence increasing due to aging populations and rising rates of diabetes and hypertension [4, 5]. Both conditions impose substantial morbidity, healthcare costs, and premature mortality [6, 7].

The coexistence of COPD and CKD is common yet underrecognized. Epidemiologic studies suggest 20%-30% of COPD patients have comorbid CKD [8, 9], and conversely, CKD patients have higher COPD prevalence than the general population [10]. This comorbidity reflects shared risk factors—smoking, aging, hypertension, diabetes—and pathophysiological links including systemic inflammation, oxidative stress, and endothelial dysfunction [11, 12]. Chronic hypoxemia from COPD activates neurohormonal pathways that accelerate renal injury [13], while CKD-associated anemia, fluid overload, and metabolic derangements worsen respiratory function [14, 15].

Patients with COPD-CKD comorbidity face worse outcomes than those with either condition alone. Observational studies report higher hospitalization rates, more frequent acute exacerbations, longer hospital stays, and increased mortality [16, 17]. CKD modifies COPD treatment responses—impaired drug clearance necessitates dose adjustments, while concerns about nephrotoxicity may limit antibiotic and nonsteroidal anti-inflammatory drug use [18]. Conversely, COPD complicates CKD management through increased infection risk, medication interactions, and diagnostic challenges (respiratory symptoms may mask uremic manifestations) [19].

Despite growing recognition of COPD-CKD interactions, national mortality trends in this population remain incompletely understood. Previous studies examined COPD and CKD mortality independently [20, 21], but few investigated trends specifically among patients with both conditions. Understanding temporal patterns and identifying high-risk subgroups is essential for resource allocation, intervention development, and policy formulation.

Using the CDC Wide-Ranging Online Data for Epidemiologic Research (WONDER) system, we conducted a comprehensive 22-year analysis of COPD-related mortality among US adults with CKD. We aimed to: (1) characterize overall temporal trends from 1999 to 2020; (2) identify disparities by sex, age, race/ethnicity, urbanization, and geography; and (3) inform clinical and public health strategies to reduce mortality in this high-risk population.

## Materials and methods

### Study setting and population

This population-based mortality study analyzed death certificate data from the CDC Wide-Ranging Online Data for Epidemiologic Research (WONDER) Multiple Cause of Death database [22] for adults aged ≥ 25 years during 1999–2020. Adults aged 25 years and older were included because COPD- and CKD-related deaths are rare before this age and because 25 years is a conventional lower bound for adult chronic-disease mortality surveillance; consistent with this, deaths below 45 years were negligible in our data (see Results). We identified deaths with chronic obstructive pulmonary disease (ICD-10 codes J40-J44) [23] as the underlying cause and chronic kidney disease (N18) [24] listed as a contributing cause, following standard CDC mortality surveillance definitions [25, 26]. This case definition therefore captures deaths co-mentioning COPD and CKD on the death certificate—that is, decedents for whom COPD was the certified underlying cause and CKD a contributing cause—rather than all individuals with coexisting COPD and CKD. As this study used deidentified public data, institutional review board approval was not required. Reporting followed STROBE guidelines [27].

### Data abstraction

Deaths were stratified by sex (male/female), race/ethnicity (Hispanic, Non-Hispanic White, Non-Hispanic Black, Non-Hispanic Other), age groups (25–34, 35–44, 45–54, 55–64, 65–74, 75–84, 85 + years), urbanization (metropolitan/nonmetropolitan per 2013 NCHS classification [28, 29]), census region (Northeast, Midwest, South, West [30]), and state.

### Statistical analysis

Crude mortality rates and age-adjusted mortality rates (AAMRs) per 100,000 population were calculated using the 2000 US standard population. For age-specific analyses, crude rates were used as they were already age-stratified. Because annual death counts for adults aged 25–34 and 35–44 years were below 20 in most years, the corresponding rates were unreliable by CDC WONDER standards; these age groups were therefore excluded, and age-stratified analyses were restricted to adults aged ≥ 45 years. Temporal trends were assessed via Joinpoint regression (Version 5.2.0) [31], yielding annual percent changes (APCs) and average annual percent changes (AAPCs) with 95% confidence intervals. The number and location of joinpoints were selected by the grid-search method with a Monte Carlo permutation test (4499 permutations; overall significance level 0.05); a maximum of five joinpoints was permitted for the 22-year series, with a minimum of two observations from each joinpoint to the ends of the series and between adjacent joinpoints (software defaults). For most strata the best-fitting model contained no joinpoints (a single 1999–2020 trend); the exception was the non-Hispanic Black group, for which two joinpoints (in 2008 and 2011) were identified. Statistical significance was set at *p* < 0.05. To evaluate the potential influence of the COVID-19 pandemic, a sensitivity analysis was conducted by re-estimating the overall temporal trend after excluding 2020, using weighted log-linear regression of the annual age-adjusted rates. State-level variations were visualized using heat maps displaying total deaths, AAMRs, percent change (1999–2020), and AAPCs. Because sub-national data cannot be retrieved through the CDC WONDER application programming interface under NCHS confidentiality policy, state-level estimates were obtained directly from the CDC WONDER web interface. Total deaths and age-adjusted rates per state were derived by grouping results by state, and annual age-adjusted rates were derived by grouping results by state and year to provide the input series for state-specific Joinpoint regression. Consistent with CDC WONDER standards, counts below 10 were suppressed and rates based on fewer than 20 deaths were considered unreliable; states with insufficient reliable annual data to support stable trend estimation were not assigned an AAPC and are displayed as not available.

## Results

### Annual trends for COPD and CKD-related mortality

**Fig. 1:**
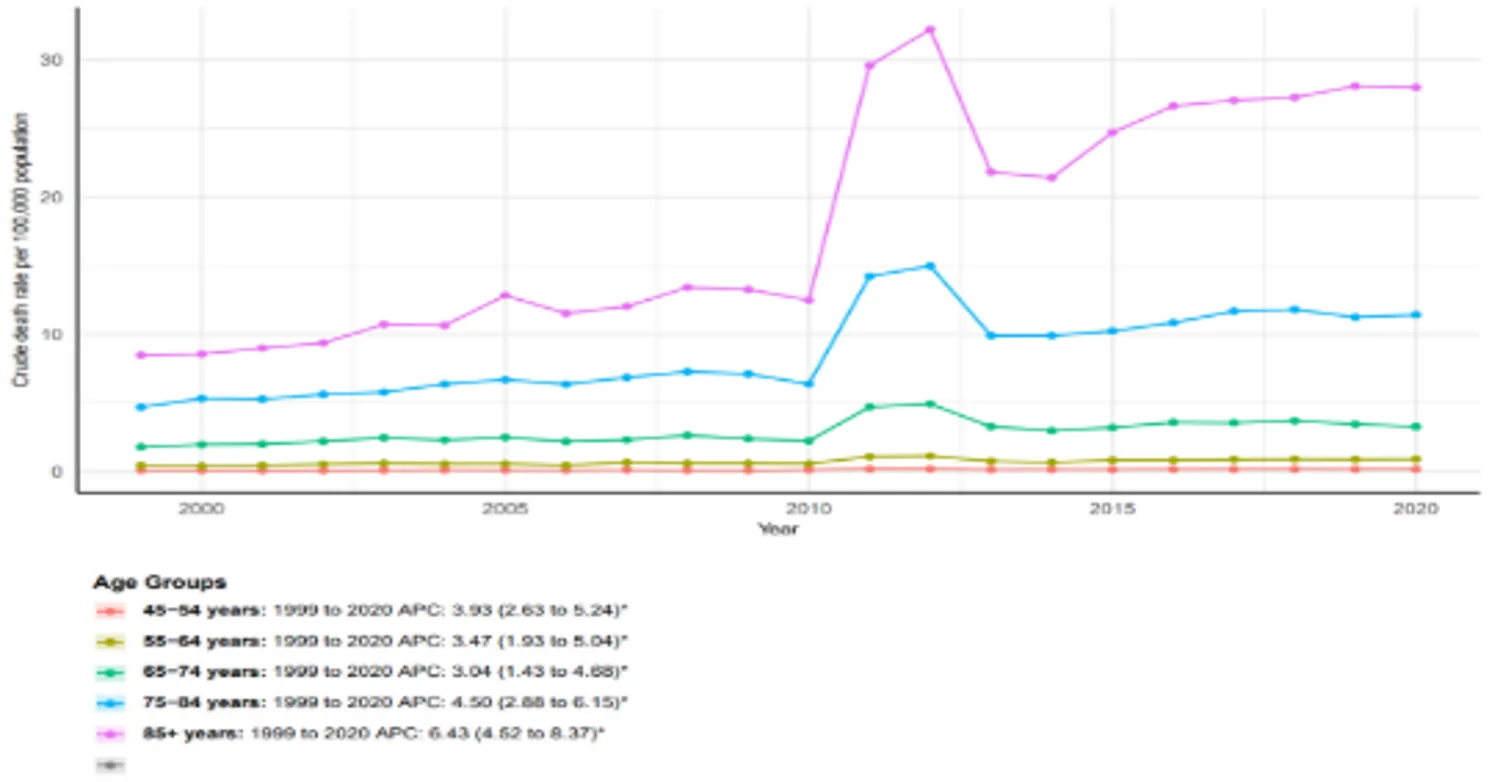
Age-stratified crude death rates per 100,000 population for COPD-related mortality among adults with chronic kidney disease in the United States from 1999 to 2020

Deaths increased from 1,405 in 1999 to 5,277 in 2020 (Table 1). The AAMR for COPD and CKD-related deaths in adults increased from 0.79 per 100,000 population (95% CI: 0.75–0.83) in 1999 to 1.96 per 100,000 population (95% CI: 1.91–2.02) in 2020. The overall AAMR rose consistently from 1999 to 2020 (AAPC: +4.71%; 95% CI: 3.06–6.38; *p* < 0.001), highlighting a substantial rise over the study period (Fig. 1; Table 1). In a sensitivity analysis excluding 2020, the overall AAPC was similar (5.06%; 95% CI: 3.40–6.76); the age-adjusted rate in 2020 (1.96 per 100,000 population) was comparable to that in 2019 (1.98), indicating that the increasing trend was not driven by the pandemic year.

**Table 1:** Mortality trends stratified by demographics and geography for COPD-related deaths among adults with chronic kidney disease in the United States, 1999–2020

Measure	Deaths_1999	Deaths_2020	percent.change	AAMR_1999	AAMR_2020	AAPC (95% CI
Both	1405	5277	275.59	0.79 (0.75 to 0.83)	1.96 (1.91 to 2.02)	4.71 (3.06 to 6.38)*
Female	523	2557	388.91	0.50 (0.46 to 0.55)	1.65 (1.58 to 1.71)	5.86 (4.07 to 7.69)*
Male	882	2720	208.39	1.32 (1.23 to 1.41)	2.41 (2.32 to 2.50)	3.42 (1.85 to 5.02)*
Midwest	336	1413	320.54	0.78 (0.70 to 0.87)	2.42 (2.30 to 2.55)	5.32 (3.73 to 6.94)*
Northeast	316	750	137.34	0.85 (0.75 to 0.94)	1.49 (1.39 to 1.60)	3.87 (2.51 to 5.26)*
South	503	2068	311.13	0.80 (0.73 to 0.88)	2.03 (1.94 to 2.12)	4.65 (2.98 to 6.36)*
West	250	1046	318.4	0.71 (0.62 to 0.80)	1.74 (1.63 to 1.84)	4.59 (2.55 to 6.67)*
Hispanic	43	219	409.3	0.57 (0.40 to 0.77)	0.94 (0.82 to 1.07)	2.36 (0.46 to 4.30)*
NH Black	28	109	289.29	0.66 (0.42 to 0.97)	0.76 (0.61 to 0.90)	-0.22 (-7.79 to 7.98)
NH Other	159	533	235.22	1.09 (0.92 to 1.26)	2.09 (1.91 to 2.28)	3.30 (1.85 to 4.77)*
NH White	1172	4407	276.02	0.78 (0.73 to 0.82)	2.14 (2.08 to 2.21)	5.25 (3.60 to 6.92)*
Metropolitan	1107	4072	267.84	0.77 (0.73 to 0.82)	1.82 (1.76 to 1.87)	4.47 (2.84 to 6.13)*
Nonmetropolitan	298	1205	304.36	0.87 (0.77 to 0.97)	2.68 (2.53 to 2.84)	5.71 (3.99 to 7.47)*
45–54 years	34	73	114.71	0.09 (0.09 to 0.09)	0.18 (0.18 to 0.18)	3.93 (2.63 to 5.24)*
55–64 years	107	385	259.81	0.45 (0.45 to 0.45)	0.91 (0.91 to 0.91)	3.47 (1.93 to 5.04)*
65–74 years	330	1065	222.73	1.79 (1.79 to 1.79)	3.27 (3.27 to 3.27)	3.04 (1.43 to 4.68)*
75–84 years	573	1879	227.92	4.69 (4.69 to 4.69)	11.42 (11.42 to 11.42)	4.50 (2.88 to 6.15)*
85 + years	352	1863	429.26	8.47 (8.47 to 8.47)	27.98 (27.98 to 27.98)	6.43 (4.52 to 8.37)*

### COPD and CKD-related mortality trends stratified by sex

**Fig. 2:**
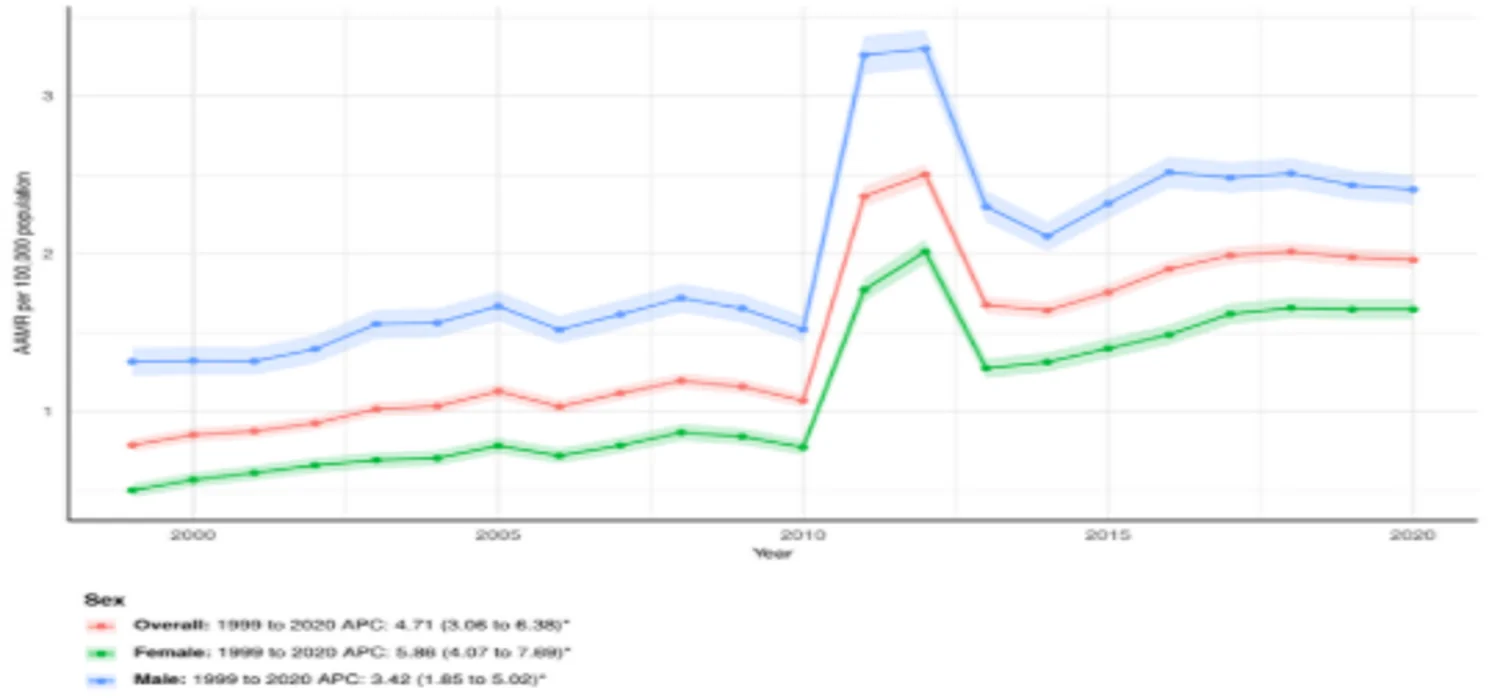
Overall and sex-stratified COPD-related AAMRs per 100,000 population among adults with chronic kidney disease in the United States from 1999 to 2020

Trends in mortalities differed by sex. The overall AAMR was 2.41 (95% CI: 2.32–2.50) for males and 1.65 (95% CI: 1.58–1.71) for females (Table 1). For males, the AAMR increased from 1.32 in 1999 to 2.41 in 2020, showing an AAPC of + 3.42% (95% CI: 1.85–5.02; *p* < 0.001). For females, the AAMR rose from 0.50 in 1999 to 1.65 in 2020, with an AAPC of + 5.86% (95% CI: 4.07–7.69; *p* < 0.001) (Fig. 2; Table 1).

### COPD and CKD-related mortality trends stratified by age

Age-stratified analyses were restricted to adults aged ≥ 45 years, for whom annual rates were reliable; younger age groups were excluded because their counts were too small to yield stable estimates (see Methods). Figure 2 displays crude death rates for these five age groups. Deaths increased across all age groups: 45–54 years (34→73, APC + 3.93%), 55–64 years (107→385, APC + 3.47%), 65–74 years (330→1,065, APC + 3.04%), 75–84 years (573→1,879, APC + 4.50%), and 85 + years (352→1,863, APC + 6.43%). The 85 + age group had the highest crude mortality rate (27.98 per 100,000 population in 2020) and fastest growth (Fig. 2; Table 1).

### COPD and CKD-related mortality trends stratified by race

The overall AAMR was highest among NH White adults (2.14; 95% CI: 2.08–2.21), followed by NH Other (2.09; 95% CI: 1.91–2.28), Hispanic adults (0.94; 95% CI: 0.82–1.07), and NH Black adults (0.76; 95% CI: 0.61–0.90) (Table 1).

**Fig. 3:**
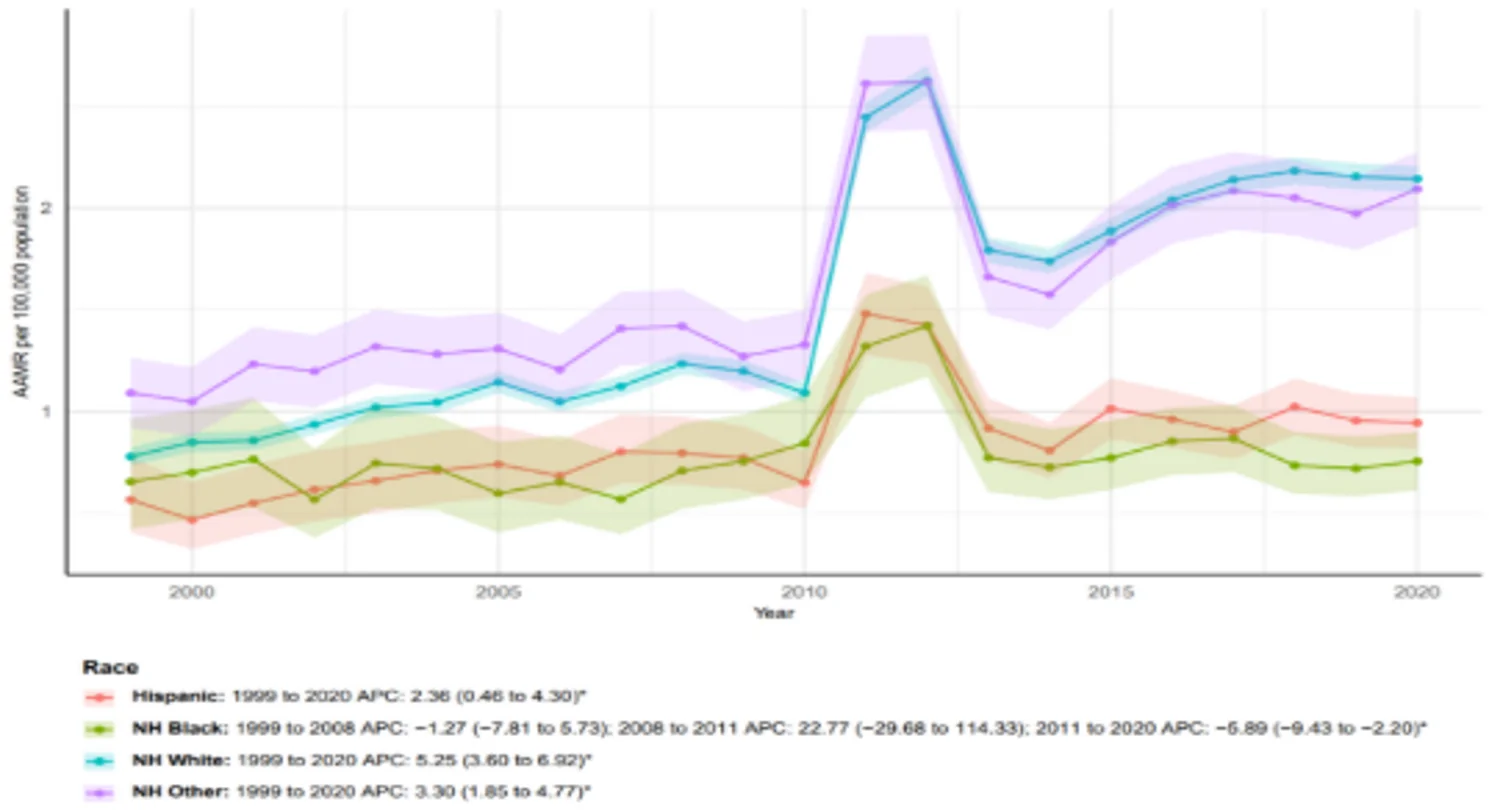
Race/ethnicity-stratified COPD-related AAMRs per 100,000 population among adults with chronic kidney disease in the United States from 1999 to 2020

NH White adults experienced a steady increase from 1999 to 2020 (AAPC: +5.25%; 95% CI: 3.60–6.92; *p* < 0.001). NH Other showed an AAPC of + 3.30% (95% CI: 1.85–4.77; *p* < 0.001). Hispanic adults had an AAPC of + 2.36% (95% CI: 0.46–4.30; *p* = 0.017). NH Black individuals showed non-linear trends: declining 1999–2008 (APC − 1.27%, not significant [NS]), volatility 2008–2011 (APC + 22.77%, NS), and significant decline 2011–2020 (APC − 5.89%, *p* < 0.05). Overall AAPC was − 0.22% (*p* = 0.96), the only group without significant increase (Fig. 3; Table 1).

### Geographic mortality patterns

#### Census regions

**Fig. 4:**
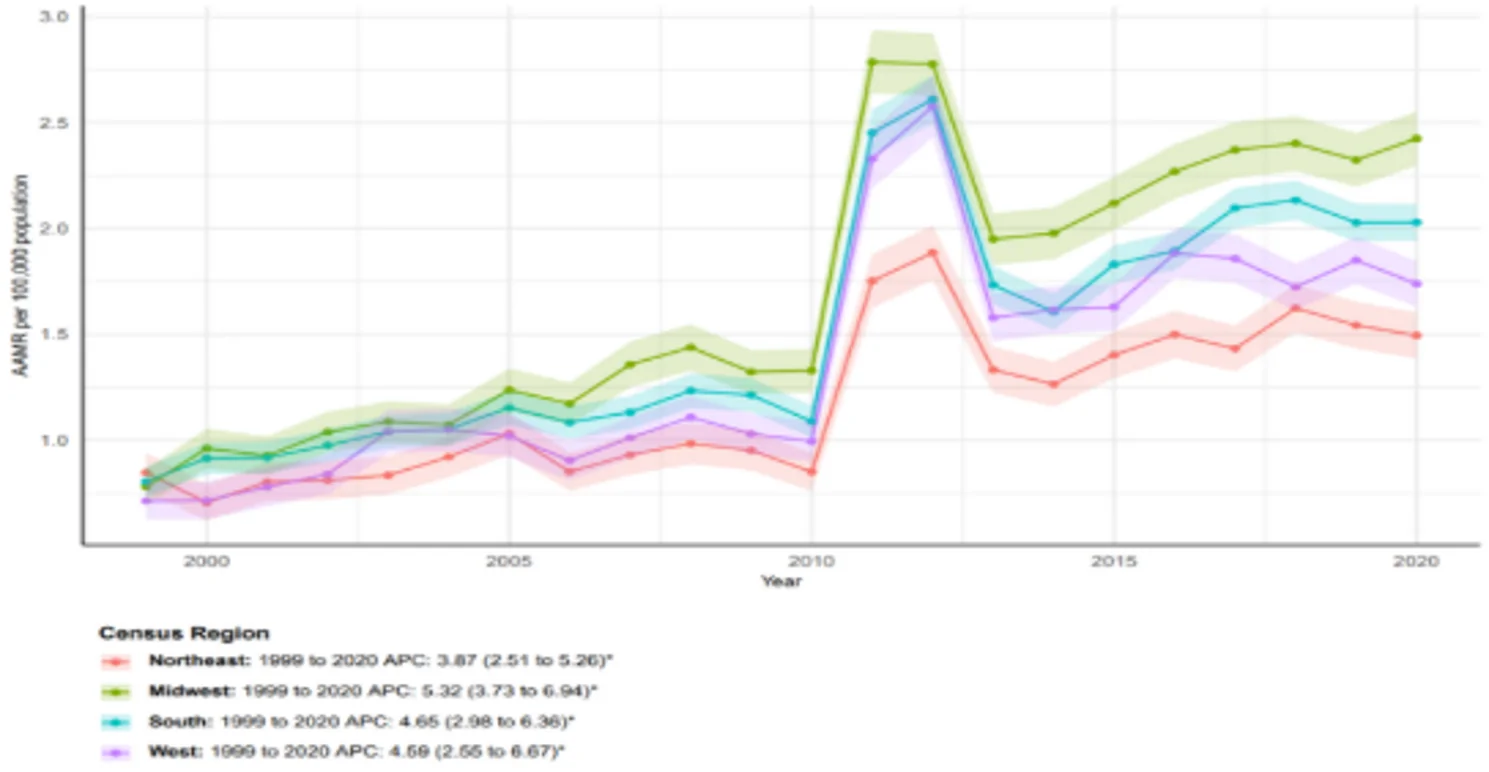
Census region-stratified COPD-related AAMRs per 100,000 population among adults with chronic kidney disease in the United States from 1999 to 2020

The Midwest had the highest AAPC (+ 5.32%) and 2020 AAMR (2.42 per 100,000 population), followed by South (+ 4.65%, 2.03 per 100,000 population), West (+ 4.59%, 1.74 per 100,000 population), and Northeast (+ 3.87%, 1.49 per 100,000 population) (Fig. 4; Table 1). A consistent geographic gradient was thus apparent, with both the 2020 age-adjusted mortality burden and its rate of increase greatest in the Midwest and progressively lower across the South, West, and Northeast; all four regional AAPCs were statistically significant (*p* < 0.001).

#### State-level variation

**Fig. 5:**
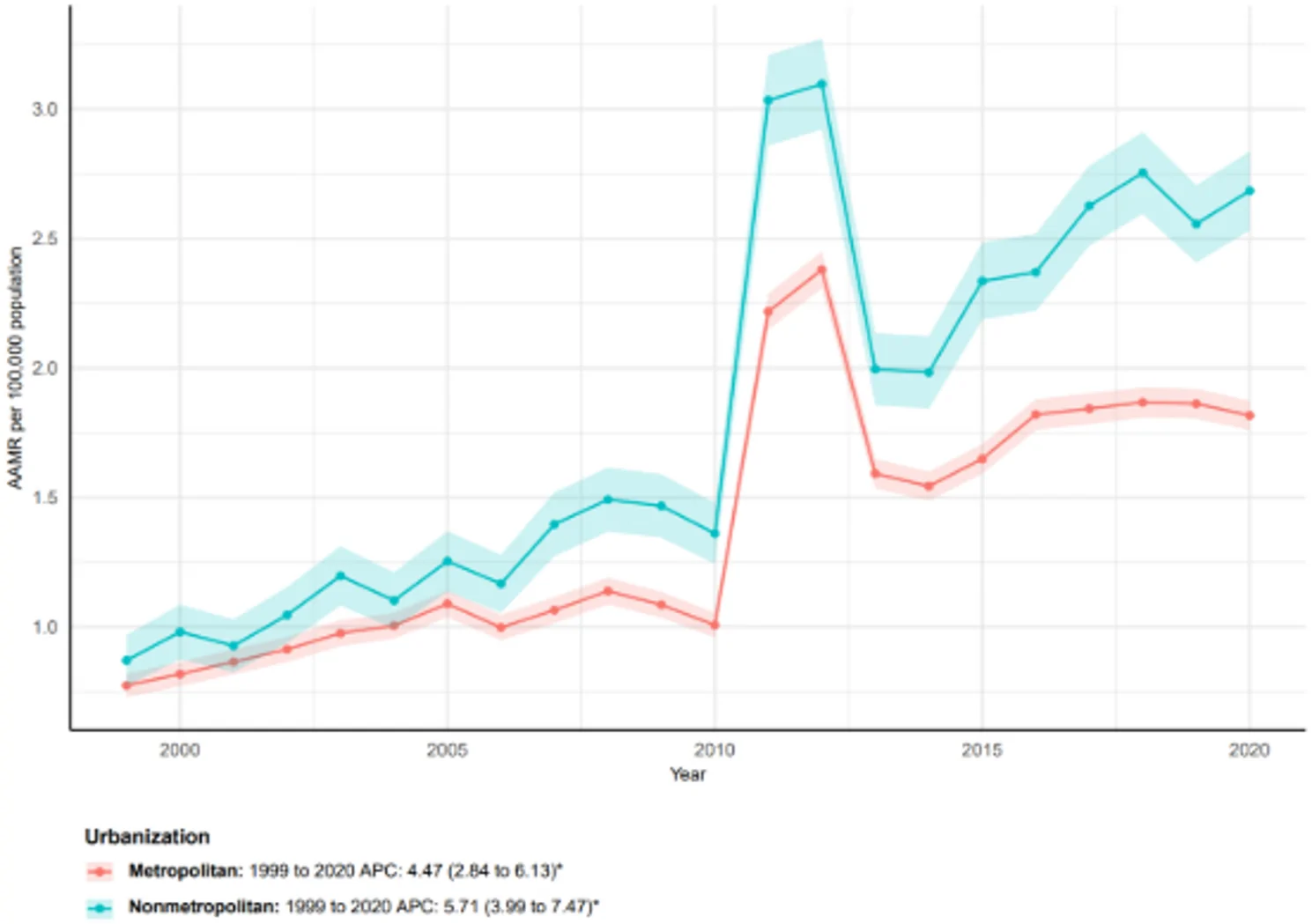
Urbanization-stratified COPD-related AAMRs per 100,000 population among adults with chronic kidney disease in the United States from 1999 to 2020

Figure 5 A shows total deaths ranged from < 25 in small states to 238–468 in populous states. Panel B reveals AAMR variation from 1.16 to 1.70 per 100,000 population (Southwest/West) to 3.15–4.40 per 100,000 population (Midwest/Appalachia). Panel C shows most states had 95–155% increases, with Nevada and South Carolina exceeding 155%. Panel D demonstrates AAPC variation from 1.5 to 3.5% (Southwest) to 6.0–7.0% (Maine, Vermont, Wisconsin, Michigan, Kentucky, West Virginia, Florida, Oregon, Utah). State-specific 2020 age-adjusted rates, AAPCs, 95% confidence intervals, and rankings for all states with estimable trends are provided in the Supplementary Data (State Summary).

### | COPD and CKD-related mortality trends stratified by urbanization

**Fig. 6:**
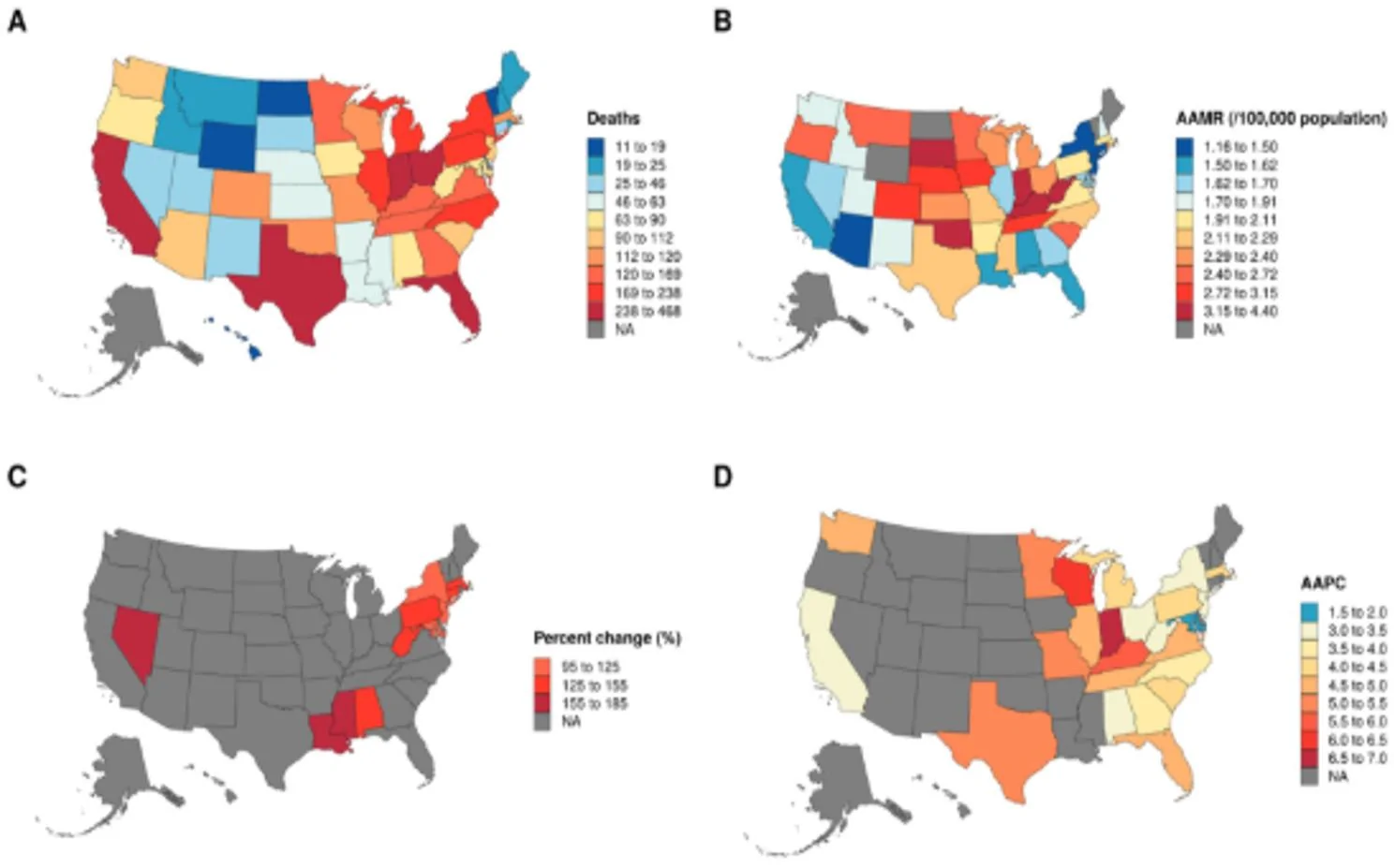
Geographic distribution of COPD-related mortality among adults with chronic kidney disease in the United States, 1999–2020. (**A**) Total death counts by state. (**B**) Age-adjusted mortality rates (AAMRs) per 100,000 population by state. (**C**) Percent change in death counts from 1999 to 2020 by state. (**D**) Average annual percent change (AAPC) in mortality rates by state

Nonmetropolitan areas exhibited a higher AAMR than metropolitan areas (Table 1). The AAMR was 2.68 (95% CI: 2.53–2.84) for nonmetropolitan areas and 1.82 (95% CI: 1.76–1.87) for metropolitan areas. Metropolitan areas had an AAPC of + 4.47% (95% CI: 2.84–6.13; *p* < 0.001), while nonmetropolitan areas had an AAPC of + 5.71% (95% CI: 3.99–7.47; *p* < 0.001) (Fig. 6; Table 1).

## Discussion

This 22-year analysis of CDC WONDER data reveals a substantial burden of COPD-related mortality among adults with CKD in the United States. Between 1999 and 2020, deaths increased 276% with an AAPC of + 4.71%, demonstrating persistent growth across all demographic subgroups. Notable disparities emerged by sex, race/ethnicity, age, and geography, with females, older adults, NH White individuals, and Midwest/Appalachian states experiencing disproportionate increases. Throughout, these findings should be read as trends in deaths certified with COPD as the underlying cause and CKD as a contributing cause; they characterize co-mentioned COPD–CKD mortality on death certificates rather than mortality among the entire population living with both conditions, and the interpretations below are framed accordingly. Relative increases (AAPCs) are also distinguished from absolute rates throughout, because large percentage increases can arise in subgroups with low baseline mortality; for example, females showed a faster relative increase than males (AAPC 5.86% vs. 3.42%) yet retained a lower absolute age-adjusted rate (1.65 vs. 2.41 per 100,000 population in 2020).

### Cardiopulmonary-renal interactions

The co-occurrence of COPD and CKD reflects shared pathophysiological mechanisms. Chronic hypoxemia from COPD impairs renal perfusion and activates the renin-angiotensin-aldosterone system, accelerating CKD progression [10]. Conversely, CKD-associated fluid overload, anemia, and uremia exacerbate respiratory dysfunction and increase susceptibility to respiratory infections [32]. Systemic inflammation, oxidative stress, and endothelial dysfunction are common to both conditions [33, 34], creating a self-reinforcing cycle of organ damage. This multiorgan involvement likely explains the elevated mortality observed in our cohort, where patients face compounded risks from both pulmonary and renal failure. These pulmonary–renal crosstalk pathways—encompassing systemic inflammation, hypoxemia, neurohormonal activation, and endothelial dysfunction—have been detailed in recent reviews [35, 36].

### Rising mortality trends

The sustained 4.71% annual increase in COPD-CKD mortality parallels trends in both conditions independently. COPD mortality has risen in recent decades despite declining smoking rates, attributed to aging populations, increasing biomass fuel exposure, and air pollution [37, 38]. Similarly, CKD prevalence has grown due to rising diabetes and hypertension rates [7]. Our findings suggest these epidemics intersect with multiplicative rather than additive effects on mortality.

The acceleration from 2018 to 2020 warrants attention. While broader trends reflect demographic aging and disease prevalence increases, the 2020 spike likely reflects COVID-19 impacts. Patients with COPD-CKD comorbidity faced heightened vulnerability to severe COVID-19 complications [39], and pandemic-related healthcare disruptions may have delayed routine disease management. Additionally, increased medical scrutiny during the pandemic may have improved death certificate coding accuracy [40].

### Sex disparities

Females demonstrated faster mortality increases (AAPC + 5.86%) despite lower absolute rates than males. This paradox reflects changing smoking patterns—while male smoking rates declined earlier, female smoking prevalence remained elevated through the 1990–2000 s, creating a cohort effect now manifesting in mortality [41, 42]. Additionally, females may have greater susceptibility to COPD at equivalent smoking exposures due to smaller airway dimensions and sex-hormone effects on airway biology and on the metabolism of tobacco-smoke constituents [43]. Diagnostic delays in females, where COPD symptoms are often attributed to aging or anxiety, may contribute to more advanced disease at diagnosis [44]. The loss of estrogen’s renoprotective effects post-menopause may also accelerate CKD progression in females with COPD [45]. These observations are not contradictory but reflect the organ-specific actions of estrogen: in the airways, estrogen-mediated bioactivation of tobacco-smoke constituents and modulation of airway inflammation may heighten susceptibility to smoking-related airflow limitation, whereas in the kidney estrogen is predominantly protective, attenuating glomerulosclerosis and interstitial fibrosis through anti-inflammatory and hemodynamic mechanisms. Tissue-specific differences in hormone-receptor distribution and downstream signaling thus allow the same hormonal milieu to be deleterious in the lung yet beneficial in the kidney, so that its post-menopausal decline removes a renoprotective influence while the cumulative pulmonary effects of prior exposure persist.

### Age-specific patterns

The exponential increase in mortality with age—from APC + 3.04% in 65–74 year-olds to + 6.43% in those 85+—reflects cumulative disease burden, declining physiologic reserve, and reduced tolerance for acute decompensations [46]. Older adults with COPD-CKD comorbidity face challenges from polypharmacy, frailty, and limited rehabilitation potential [47]. The near-absence of deaths in adults < 45 years suggests COPD-CKD mortality primarily affects those with decades of exposure to risk factors, though early-onset casesmay reflect genetic predisposition; however, this possibility is speculative and cannot be evaluated with death-certificate data that lack individual clinical information.

### Racial and ethnic disparities

NH White individuals had the highest mortality rates and fastest increases (AAPC + 5.25%), consistent with higher COPD prevalence in this population [48, 49]. Genetic factors may contribute [50]. Higher historical smoking rates and greater occupational exposures (mining, agriculture) in predominantly White rural areas likely play roles [51].

The unique pattern in NH Black individuals—with non-linear trends and an overall non-significant AAPC—merits discussion. While this population has lower COPD prevalence despite higher smoking rates [52], possible explanations include: (1) survival bias, where NH Black individuals with severe COPD may die before developing advanced CKD; (2) differences in COPD phenotypes [53]; (3) improved CKD management in this population through initiatives targeting diabetic nephropathy [54]; or (4) coding inconsistencies on death certificates. The decline from 2011 to 2020 (APC − 5.89%) coincides with increased nephrology care access following the Affordable Care Act; however, because the database contains no information on healthcare access or insurance coverage, any link to the Affordable Care Act is a temporal coincidence only and remains speculative and hypothesis-generating.

Hispanic individuals maintained the lowest mortality rates, potentially reflecting the “Hispanic paradox,” healthier lifestyle factors, or younger population demographics [55]. However, their significant AAPC (+ 2.36%) indicates rising burden requiring attention.

### Geographic variations

The Midwest’s highest mortality rates and fastest increases (AAPC + 5.32%, 2020 AAMR 2.42 per 100,000 population) align with regional characteristics: high smoking prevalence, coal mining and agricultural occupational exposures, industrial air pollution, and limited healthcare access in rural areas [56, 57]. States like Kentucky, West Virginia, and Ohio—with historically coal-dependent economies—bear disproportionate burdens. At the opposite end of the gradient, the Northeast recorded both the lowest 2020 AAMR and the slowest increase, consistent with lower smoking prevalence, greater urbanization, and denser access to pulmonary and nephrology specialty care, while the South and West occupied intermediate positions. The resulting stepwise Midwest-to-Northeast pattern suggests that regional differences in tobacco exposure, occupational and environmental risk, and healthcare access—rather than population age structure alone, given that rates were age-adjusted—drive the observed geographic disparities.

A significant difference in AAMRs was observed across states, with rates ranging from 1.16 to 1.70 per 100,000 population (Southwest/West) to 3.15–4.40 per 100,000 population (Midwest/Appalachia) (Fig. 6). States in the top 90th percentile—Maine, Vermont, Wisconsin, Michigan, Kentucky, West Virginia, Florida, Oregon, and Utah—had almost triple the AAMRs compared with states in the lower 10th percentile, namely Nevada, Arizona, California, and Texas. The Southwest’s lower rates may reflect migration of healthier individuals, warmer climates reducing COPD exacerbations, and lower smoking rates [58]. These influences—including warmer climate and selective migration of healthier individuals—are hypothesized rather than measured, as the database captures neither environmental exposures nor residential history. However, rapid growth in Nevada and Arizona suggests emerging problems requiring intervention.

State-level variation (AAPC range 1.5-7.0%) indicates modifiable factors beyond demographic composition. States with robust tobacco control, clean air policies, and integrated pulmonary-renal care models may serve as blueprints for high-burden regions [59].

### Rural-urban disparities

Although nonmetropolitan areas had a higher growth rate (AAPC + 5.71% vs. + 4.47% for metropolitan areas), they maintained 47% higher 2020 mortality than metropolitan areas. Rural populations face multiple disadvantages: higher smoking and obesity rates, occupational exposures (farming, mining), provider shortages (particularly pulmonologists and nephrologists), delayed diagnoses, and barriers to specialty care [60, 61]. Rural patients are less likely to receive guideline-concordant COPD therapies (inhaled corticosteroids, long-acting bronchodilators) or CKD management (renin-angiotensin system inhibitors, sodium-glucose cotransporter-2 inhibitors) [62, 63]. Telemedicine expansion and mobile specialty clinics may help bridge these gaps.

### Clinical and policy implications

These findings have several implications. First, integrated care models coordinating pulmonology and nephrology are needed for COPD-CKD patients, who often receive fragmented care [64]. Second, given the age-specific burden, geriatric assessment including frailty screening should guide treatment intensity in older adults [65]. Third, addressing modifiable risk factors—smoking cessation, air quality improvement, vaccination against respiratory pathogens, optimal management of hypertension and diabetes—remains paramount [66, 67]. Fourth, rural health infrastructure investment is critical to reducing geographic disparities.

Clinicians should maintain high suspicion for CKD in COPD patients and vice versa, with routine screening using creatinine, estimated glomerular filtration rate, and urinary albumin [68]. Early identification enables intervention before irreversible organ damage occurs. Medication reconciliation is essential, as drugs commonly used in one condition may worsen the other (e.g., nonsteroidal anti-inflammatory drugs exacerbating CKD, beta-blockers worsening COPD) [69].

Public health efforts should target high-risk populations identified in this study: females, older adults, NH White individuals, and residents of Midwest/Appalachian states and rural areas. Tailored interventions addressing region-specific risk factors (occupational exposures in mining states, agricultural dust in rural areas) may yield benefits.

An important consideration in interpreting these trends is that the observed increase may partly reflect changes over time in how CKD is recognized, documented, and coded on death certificates rather than a true change in disease burden. Awareness and laboratory ascertainment of CKD expanded substantially over the study period, and successive clinical guidelines heightened recognition of CKD as a comorbid and contributing condition; certifying physicians may therefore have recorded CKD as a contributing cause more frequently in later years. Such improved ascertainment would inflate the apparent upward trend—most plausibly contributing to the abrupt 2010–2011 discontinuity discussed below—and part of the rise may represent better capture of co-mentioned COPD–CKD mortality rather than a genuine increase in its occurrence. Because certification and coding practices may also have evolved unevenly across regions and populations, subgroup comparisons could be affected if documentation improved differentially. For these reasons, the magnitude of the increases reported here should be regarded as an upper bound, and the temporal and demographic patterns interpreted with corresponding caution.

### Limitations

This study has limitations. First, death certificate data may underreport or misclassify CKD as a contributing cause, particularly if not clinically recognized or deemed irrelevant by certifying physicians [70]. Second, we could not assess disease severity, treatment received, or socioeconomic factors beyond urbanization. Third, we lacked data on smoking history, occupational exposures, or comorbidities beyond COPD and CKD. Fourth, ecological analysis of state-level data cannot establish individual-level causation. Fifth, ICD-10 coding changes over time may introduce temporal artifacts, though our use of consistent codes (J40-J44, N18) throughout minimizes this concern. Relatedly, the abrupt rise in deaths between 2010 and 2011—more than doubling within a single year—is unlikely to represent a true change in mortality and more plausibly reflects shifts in the reporting and coding of CKD as a contributing cause on death certificates rather than a change in CKD incidence; the Joinpoint segments spanning this period, most notably the transient increase observed among NH Black adults, should therefore be interpreted with particular caution. Finally, although 2020 coincided with the COVID-19 pandemic, the age-adjusted rate in that year was comparable to 2019, and the sensitivity analysis excluding 2020 yielded a similar overall trend (AAPC 5.06%), indicating that the pandemic year did not materially drive the observed long-term increase; nonetheless, pandemic-related disruptions to healthcare use and death certification in 2020 warrant some caution in interpreting that single year. Because individual-level information on smoking, COPD severity, CKD stage, socioeconomic status, healthcare access, environmental exposures, treatment, and comorbidities was unavailable, the mechanisms proposed above to explain the demographic and geographic disparities—including survival bias, genetic susceptibility, healthcare-policy (e.g., Affordable Care Act) effects, and climate-related influences—should be regarded as hypotheses rather than established causes. More broadly, as a descriptive analysis of death-certificate data, this study identifies associations and temporal patterns and cannot establish causal relationships.

### Future directions

Future research should examine: (1) mechanisms linking COPD and CKD progression using longitudinal cohorts with biomarker assessments; (2) effectiveness of integrated care models on mortality and quality of life; (3) optimal medication regimens for COPD-CKD comorbidity, particularly novel agents (triple inhaled therapy for COPD, SGLT2 inhibitors for CKD) [71, 72]; (4) implementation strategies for telemedicine-based specialty care in rural areas; and (5) policy evaluations of tobacco control and air quality regulations on COPD-CKD mortality.

## Conclusion

COPD-related mortality among adults with CKD increased substantially from 1999 to 2020, with significant disparities by sex, age, race/ethnicity, and geography. Females, older adults, NH White individuals, Midwest/Appalachian states, and rural areas experienced disproportionate burdens. These findings underscore the need for integrated pulmonary-renal care, targeted interventions for high-risk populations, and policies addressing tobacco use, air quality, and rural healthcare access. As the US population ages and chronic disease prevalence rises, the intersection of COPD and CKD will increasingly challenge healthcare systems, necessitating proactive strategies to mitigate mortality. These conclusions apply specifically to deaths certified with COPD as the underlying cause and CKD as a contributing cause, and should not be generalized to all individuals with coexisting COPD and CKD.

## Supplementary Information

Supplementary Material 1.

## Data Availability

The data that support the findings of this study are available in National Center for Health Statistics at [https://wonder.cdc.gov/mcd.html](https:/wonder.cdc.gov/mcd.html) . These data were derived from the following resources available in the public domain: CDC WONDER, [https://wonder.cdc.gov/](https:/wonder.cdc.gov) .

## Abbreviations

AAMR: Age-adjusted Mortality Rate
AAPC: Average Annual Percent change
APC: Annual Percent Change
CDC: Centers for Disease Control and Prevention
CI: Confidence Interval
CKD: Chronic Kidney Disease
COPD: Chronic Obstructive Pulmonary Disease
COVID-19: Coronavirus Disease 2019
ICD-10: International Classification of Diseases, Tenth Revision
KDIGO: Kidney Disease: Improving Global Outcomes
NCHS: National Center for Health Statistics
NH: non-Hispanic
NS: Not Significant
SE: Standard Error
STROBE: Strengthening the Reporting of Observational Studies in Epidemiology
US: United States
WONDER: Wide-ranging Online Data for Epidemiologic Research
